# Mitochondrial DNA in Residual Leukemia Cells in Cerebrospinal Fluid in Children with Acute Lymphoblastic Leukemia

**DOI:** 10.4021/jocmr443w

**Published:** 2010-10-11

**Authors:** Kathryn Egan, Ian Kusao, David Troelstrup, Melissa Agsalda, Bruce Shiramizu

**Affiliations:** a Department of Child & Adolescent Psychiatry, Tripler Army Medical Center, Hawaii, USA; b Pediatric Hematology/Oncology Division, John A. Burns School of Medicine, University of Hawaii, USA

## Abstract

This feasibility study was designed to assess the ability to measure mitochondrial DNA (mtDNA) in cerebrospinal fluid (CSF) cells that contributed to minimal disease/persistent or residual disease (MD/PRD) from children with acute lymphoblastic leukemia (ALL). Increase in mtDNA copies in cancer cells has been suggested to play a role in MD/PRD. CSF as well as blood specimens from 6 children were assayed for MD/PRD and mtDNA copy numbers by quantitative real-time polymerase chain reaction. Of 7 MD/PRD-positive specimens, 6 had increased mtDNA copy numbers; while 11 MD/PRD-negative specimens had no increase in mtDNA copy numbers, p < 0.003. This is the first proof-of-concept study to measure mtDNA copy numbers in MD/PRD-positive CSF specimens from children with ALL. Increase of mtDNA copy numbers in MD/PRD childhood ALL cells and its significance as a mechanism for recurrence requires further investigation.

**Keywords:**

Minimal residual disease; Acute lymphoblastic leukemia; Central nervous system; Cerebrospinal fluid; Mitochondria

## Introduction

The presence of minimal disease/persistent or residual disease (MD/PRD) is associated with poor prognosis in childhood acute lymphoblastic leukemia (ALL) [1-6]. In identifying presence of MD/PRD as a risk factor, Schultz et al developed an algorithm using MD to help guide treatment for B-precursor ALL [7]. The significance of MD/PRD presence during ALL therapy lies in the possibility that residual leukemic cells could represent a survival phenotype leading to recurrence of disease. Recent studies by Kusao et al suggest that malignant cells might be able to overcome chemotoxicity by increasing the number of mitochondrial DNA (mtDNA) which could translate to increase energy production leading to malignant cell survival [8, 9]. From clinical specimens, those which were positive for MD/PRD had increased mtDNA copies per cell compared to specimens which were negative for MD/PRD [8]. Kusao et al hypothesized that surviving malignant cells exposed to chemotherapy would compensate by increasing mtDNA copies which was demonstrated through in vitro experiments [8].

Assessing MD/PRD in the cerebrospinal fluid (CSF) provides additional challenges due to the relatively small number of cells. However, when central nervous system (CNS) relapse is diagnosed from analyzing CSF cells, the percentages of CSF lymphoblasts is relatively high compared to analyses of peripheral blood lymphoblast percentages. Thus on a per-cell basis, recognizing the opportunity to study mtDNA copies in CSF lymphoblasts provided the mechanism for the proof-of-concept study to determine if mtDNA copy numbers could be measured in CSF lymphoblasts from clinical specimens positive for MD/PRD. We posit that CSF and peripheral blood specimens, which are positive for MD/PRD, would have increased mtDNA copy numbers compared to MD/PRD-negative specimens.

## Patients and Methods

### Patients and specimens

CSF cells and peripheral blood mononuclear cells (PBMC) were obtained from a tissue repository which had previously processed and stored CSF and blood from pediatric ALL cases without patient identifiers as per guidelines by the University of Hawaii Institutional Review Board. Cases were selected from the repository from which diagnostic and follow-up specimens were available for CSF; and two consecutive follow-up specimens available for PBMC; all from children diagnosed with pre-B-cell ALL. Age, gender, and limited clinical data were available for each case.

### Minimal residual disease and mitochondrial DNA assessments

The PBMC and CSF samples were previously assessed for MD/PRD [10, 11]. Briefly, the strategy for MD/PRD identification used patient-specific primers (PSP) from consensus primers of the third complementary determining region of the immunoglobulin heavy chain region and T-cell receptor-delta/gamma regions [10-12]. The identified PSP from the diagnostic specimen was then used as a marker to assess MD/PRD in follow-up specimens. The sensitivity of the MD/PRD assay was 1 in 100,000 cells.

All MD/PRD-positive and MD/PRD-negative specimens from the repository were repurified by ethanol precipitation, reconstituted in Tris-HCl-EDTA buffer, and found to be adequate in quantity and quality by UV spectrophotometry. Using techniques previously established in the laboratory, a multiplex real-time PCR assay was performed on each DNA specimen to amplify a Fas ligand nuclear region and a region of the mtDNA [8]. Briefly, dilutions of a control plasmid ranging from 10^2^ copies to 10^6^ copies containing a specific portion of mtDNA and the genomic sequence were prepared to establish the standard curves. The mitochondrial primers (forward: CAC AGA AGC TGC CAT CAA GTA; reverse: CCG GAG AGT ATA TTG TTG AAG AG) amplified the mtDNA region encoding the NADH dehydrogenase subunit 2, while the genomic primers (forward: GGC TCT GTG AGG GAT ATA AAG ACA; reverse: CAA ACC ACC CGA GCA ACTAAT CT) amplified the encoding region for Fas ligand. DNA from the specimens and appropriate positive/negative controls were set up in either duplicate or triplicate with each reaction containing iQ SYBR Green Supermix, 10 pmol forward primer and 10 pmol reverse primer with cycling parameters: 95^o^C for 1 min; 40 cycles of 95^o^C for 30 sec; 58.9^o^C for 40 sec; 72^o^C for 4 min; and 50^o^C for 30 sec. At the conclusion of the PCR, melt curve data were collected from a starting temperature of 65^o^C, with 0.5^o^C incremental increases every 5 sec for duration of 70 cycles. Copy numbers of mtDNA were obtained as previously reported with the average mtDNA copy number in PBMC or non-malignant cells ranging from 60-100 copies per cell [13].

### Statistical analyses

The Mann-Whitney test was used to determine the associations between the mtDNA copy numbers/cell in the MD/PRD-negative and MD/PRD-positive specimens. Statistical analyses were performed using SPSS, Version 16 (Chicago, IL).

## Results

**Figure 1.**
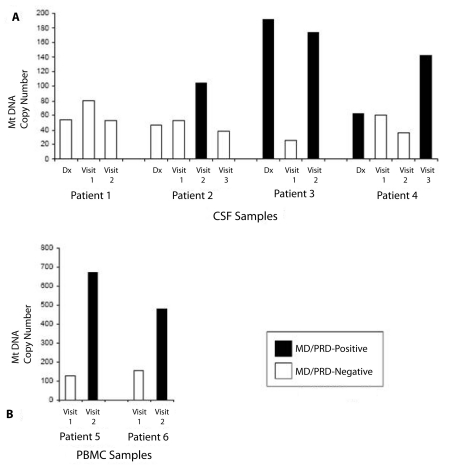
Relationship of mtDNA copy numbers/cell and positive/negative-minimal disease/persistent or residual disease (MD/PRD) Status. **A)** Cerebrospinal fluid (CSF) status of Patients 1 - 4 demonstrating relationship of MD/PRD-positive (black bar) and MD/PRD-negative (white bar) status with mtDNA copy number/cell (Y-axis). **B)** Peripheral blood mononuclear cells (PBMC) status of Patients 5 - 4 demonstrating relationship of MD/PRD-positive (black bar) and MD/PRD-negative (white bar) status with mtDNA copy number/cell (Y-axis). mtDNA copy numbers/cell for control or non-malignant cells range from 40 - 99 copies/cell [8].

Fourteen CSF samples from four children diagnosed with B-cell ALL and four additional PBMC specimens from two children with the same diagnoses were available from the tissue repository which comprised of diagnostic and follow-up specimens. Gender, age, and clinical course of the children are summarized in Table 1. For the cases from which CSF were available, CSF was obtained at the time of diagnosis, one month from beginning of induction therapy (Visit 1), 4 - 6 months from the beginning of induction therapy (Visit 2), and, when possible, 10 - 12 months from the beginning of induction therapy (Visit 3) (Fig. 1). In comparison and contrast, the cases for which PBMC specimens were available, Visits 1 and 2 corresponded to one and two months post-induction therapy, respectively. Of the 14 CSF specimens, five were positive for MD/PRD (Fig. 1A). Of the 5/14 MD/PRD-positive CSF specimens, four had increased mtDNA copy numbers compared to normal mtDNA copy numbers in PBMC from normal controls and compared to MD/PRD-negative CSF specimens, p < 0.003.

**Table 1:** Subject Demographics and Clinical Characteristics with ALL

	Age (years)	Gender	Clinical Course and Characteristics
Patient 1	5	M	No CNS disease; remission at EOT
Patient 2	7	M	No CNS disease; remission at EOT
Patient 3	6	F	CNS disease at diagnosis; CNS relapse in Year 1; remission at EOT
Patient 4	7	M	CNS disease at diagnosis; CNS relapse in Year 1; remission at EOT
Patient 5	8	M	No CNS disease; bone marrow relapse at 6 months; remission at EOT
Patient 6	6	M	No CNS disease; bone marrow relapse at 8 months; remission at EOT

M: male; F: female; CNS: central nervous system; EOT: end of therapy

**Figure 2.**
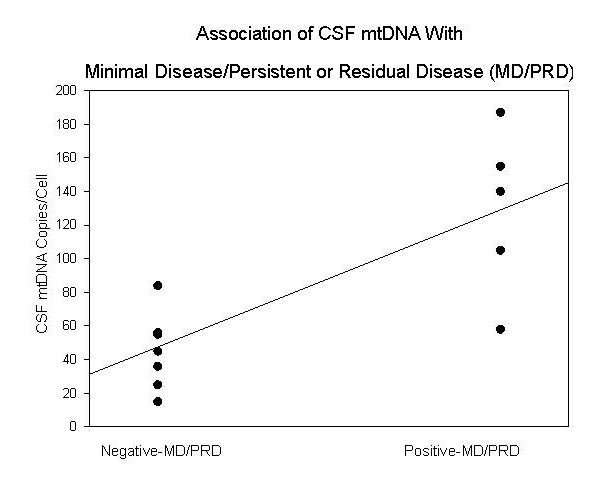
Association of CSF mtDNA copy numbers/cell with positive/negative-minimal disease/persistent or residual disease (MD/PRD) status.

Analysis of the clinical course and CSF specimens from Patient 1 showed that the child remained in clinical remission with no clinical CNS relapse; and this was reflected in the MD/PRD-negative status of the CSF diagnostic and follow-up specimens. The diagnostic and follow-up mtDNA assessment of the MD/PRD-negative CSF specimens showed that the mtDNA copy number remained relatively stable and within the normal range measured in non-malignant cells (60 - 100 copies/cell) (Fig. 1A). Similarly, Patient 2 also remained in clinical remission with no CNS disease at diagnosis or in follow-up. However, a CSF specimen from Visit 2 (4 - 6 months post-induction therapy) from Patient 2 had increase mtDNA copies/cell (104 copies/cell), which corresponded to the MD/PRD-positive status of the same specimen (Fig. 1A). The Visit 3 follow-up CSF specimen from the same patient showed clearance of the MD/PRD status with matching decrease in mtDNA copies/cell (Fig. 1A), and the patient remained in clinical remission through the end of therapy.

The CSF analyses from the next two patients showed parallel changes in MD/PRD status and mtDNA copy numbers/cell. Patients 3 and 4 both had clinical CNS disease at diagnoses; and both CSF specimens at diagnoses were MD/PRD-positive. While the diagnostic CSF for Patient 3 had high mtDNA copies/cell at diagnosis (191 copies/cell), in contrast, the diagnostic CSF from Patient 4 was low (63 copies/cell); even though both children had clinical CNS disease initially. Both Patients 3 and 4 developed CNS relapse within one year of diagnosis; and both patients had CSF specimens during the first year that were MD/PRD-positive and had high mtDNA copies/cell, 173 copies/cell at Visit 2 and 143 copies/cell at Visit 3, respectively.

To assess the relationship of mtDNA copy numbers/cell in PBMC in children with ALL, Patients 5 and 6 were selected because both children developed relapse of their leukemia within one year of diagnoses (Fig. 2). From both Patients 5 and 6, their PBMC specimens from Visits 2 were MD/PRD-positive and both had relatively high mtDNA copy numbers, 669 and 478 copies/cell, respectively; compared to the Visit 1 (one month post-induction therapy) PBMC specimens which were MD/PRD-negative and had mtDNA values of 127 and 156 copies/cell, respectively. Of interest was the fact that both Visit 2 PBMC specimens for Patients 5 and 6 had high mtDNA copies/cell in the presence of MD/PRD-negative status in the same specimens; and these two children had bone marrow relapses within the first year of therapy (Table 1). Both children underwent reinduction at the time of clinical relapse and remained in remission at the end of therapy.

## Discussion

The overall objective of this study was to determine the ability and feasibility of measuring mitochondrial DNA (mtDNA) copies per cell in CSF cells from children diagnosed with ALL in the context of the presence or absence of MD/PRD. The rationale for looking at CSF cells in childhood ALL cases in the context of mtDNA copy numbers for the current study focused on the fact that CSF lymphoblasts, when present, typically make up a higher percentage of cells in the CSF [14]. The design and methodology of the study did not sort CSF cells into subsets, i.e. lymphoblasts, therefore, the assumption was made that the mtDNA analyses from the sample represented the median copy number/cell. In this study, we report for the first time that MD/PRD-positive CSF specimens from children with ALL had high mtDNA copy numbers/cell compared to MD/PRD-negative specimens; and the possibility that MD/PRD-positive CSF specimens with an increase in mtDNA are associated with risk for CNS relapse. The data in context with other studies of mtDNA and relapse might suggest that a compensatory increase in mtDNA copy number per cell could represent a survival phenotype that leads to relapse in patients [8].

In conclusion, this study was designed as a proof-of-concept to test the feasibility of measuring mtDNA copy numbers from CSF specimens. Limitations of the study design center around the number of cases analyzed. Expanded studies are necessary to address the hypothesis that children with ALL who have MD/PRD-positive CSF specimens with high mtDNA copy numbers per cell may be at risk for CNS relapse. CNS relapse remains a major cause of morbidity in childhood ALL with treatment failure attributable to de novo or acquired resistance to a wide variety of cytotoxic drugs such as multi drug resistance [15, 16]. Additional factors associated with cell survival and phenotype leading to increase in mtDNA may also play a role in CNS relapse [8, 9]. In summary, this feasibility study warrants expanded analyses of additional children with ALL to determine the role that increased mtDNA copies in MD/PRD-positive cells in CSF and PBMC play in relapse disease.
